# Left distal ureter leiomyosarcoma: a case report

**DOI:** 10.1093/jscr/rjac501

**Published:** 2022-11-09

**Authors:** C Gan, A Attwell-Heap, A Clarke

**Affiliations:** Department of Urology, St Vincent’s Hospital Melbourne, Fitzroy, Victoria, Australia; Department of Urology, St Vincent’s Hospital Melbourne, Fitzroy, Victoria, Australia; Department of Urology, St Vincent’s Hospital Melbourne, Fitzroy, Victoria, Australia

## Abstract

Primary leiomyosarcoma of the ureter is an extremely rare, aggressive malignancy of the urinary tract. This report describes a case of primary leiomyosarcoma of the distal left ureter in a middle-aged male, with no tumor recurrence achieved following resection and end-to-end ureteroureterostomy.

## INTRODUCTION

Our patient was diagnosed with left distal ureter leiomyosarcoma following workup of an unexplained left hydroureteronephrosis on ultrasound. Wide excision was not required to achieve clear margins and a cure for this patient.

## CASE PRESENTATION

Case PD is a 59-year-old man initially admitted for left epididymo-orchitis, treated with a 6-week course of oral ciprofloxacin with resolution on repeat testicular ultrasound.

His past medical history includes bipolar disorder on lithium, methamphetamine use and sexually transmitted chlamydial infections. He does not smoke.

A renal tract ultrasound 6-week post-admission detected an unexplained 14-mm left hydroureter and hydronephrosis. An uncomplicated prostatomegaly of 38 cc was noted. His estimated glomerular filtration rate had also dropped from 78 to 56.

**Figure 1:**
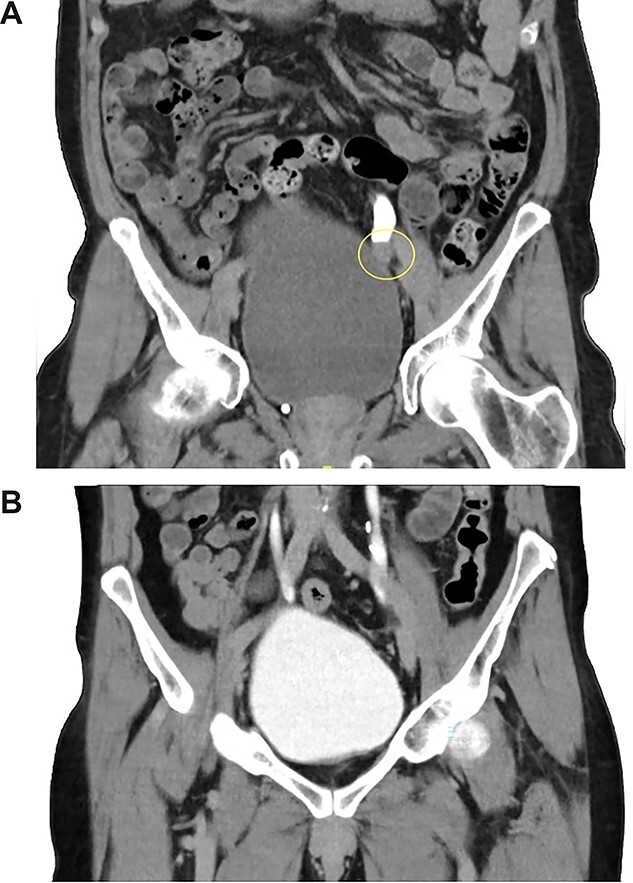
(**A**, **B**) CT-IVP before the surgical excision and 6-month post-operation.

Computed tomography-intravenous pyelogram (CT-IVP) revealed a 1.7-cm intraluminal mass within the left distal ureter that extends beyond the ureteric lumen into peri-ureteric fat, with moderate left hydroureteronephrosis (Fig. 1A).

He was readmitted for a left retrograde pyelogram, ureteroscopy and washings. No filling defect, or luminal lesion was noted intraoperatively, although the ureter was tortuous. Ureteric washings were inconclusive due to insufficient cells.

Repeat CT-IVP was conducted 2 months following the initial CT-IVP. The mass had expanded to 1.9 cm with lobulated borders. Left hydroureteronephrosis persisted, however the distal left ureter remained patent. The unit X-ray meeting recommended a relook ureteroscopy, washings and biopsy. A Mag-3 Renogram with Frusemide washout revealed high grade left renal obstruction, and split function of left 41%, right 58%. A magnetic resonance imaging suggested the mass remained stable with no invasion.

**Figure 2:**
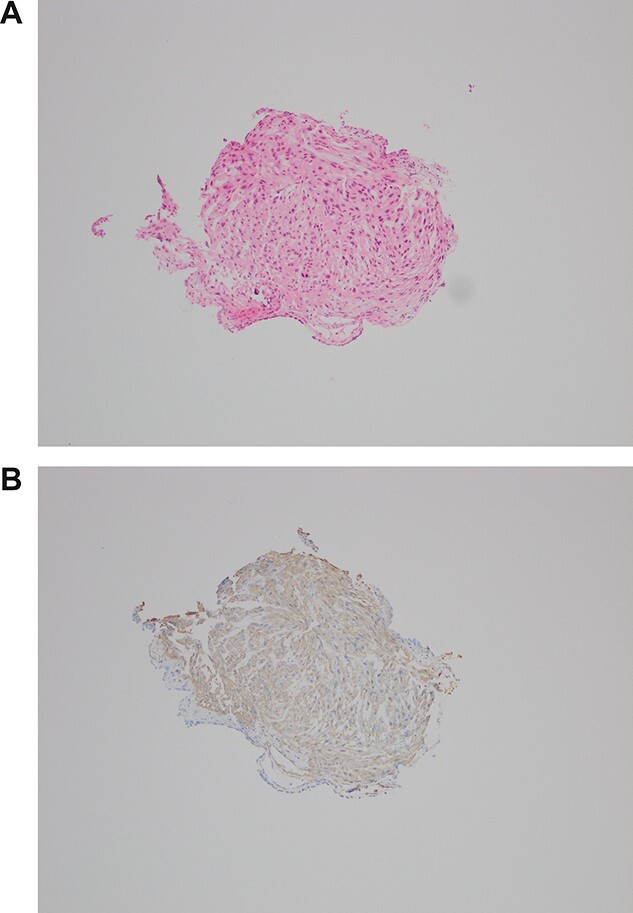
(**A**, **B**) H&E biopsy × 100 and SMA IHC—small fragment of tissue with elongate cells and moderate nuclear pleomorphism. Flattened lining could be urothelium (lower left corner). Positive for smooth muscle antigen on immunohistochemistry, consistent with leiomyosarcoma.

Case PD returned for repeat ureteroscopy, ureteric washings, biopsy and stent insertion. A large bladder volume was noted intraoperatively. There was no papillary lesion, with compression appearing to arise externally. Biopsy was smooth muscle actin positive on immunohistochemistry, and demonstrated a smooth muscle tumor with some atypia, consistent with grade 2 leiomyosarcoma (Fig. 2).

CT chest for staging revealed no metastases, but detected an incidental 5.1-cm dilated ascending aortic aneurysm.

**Figure 3:**
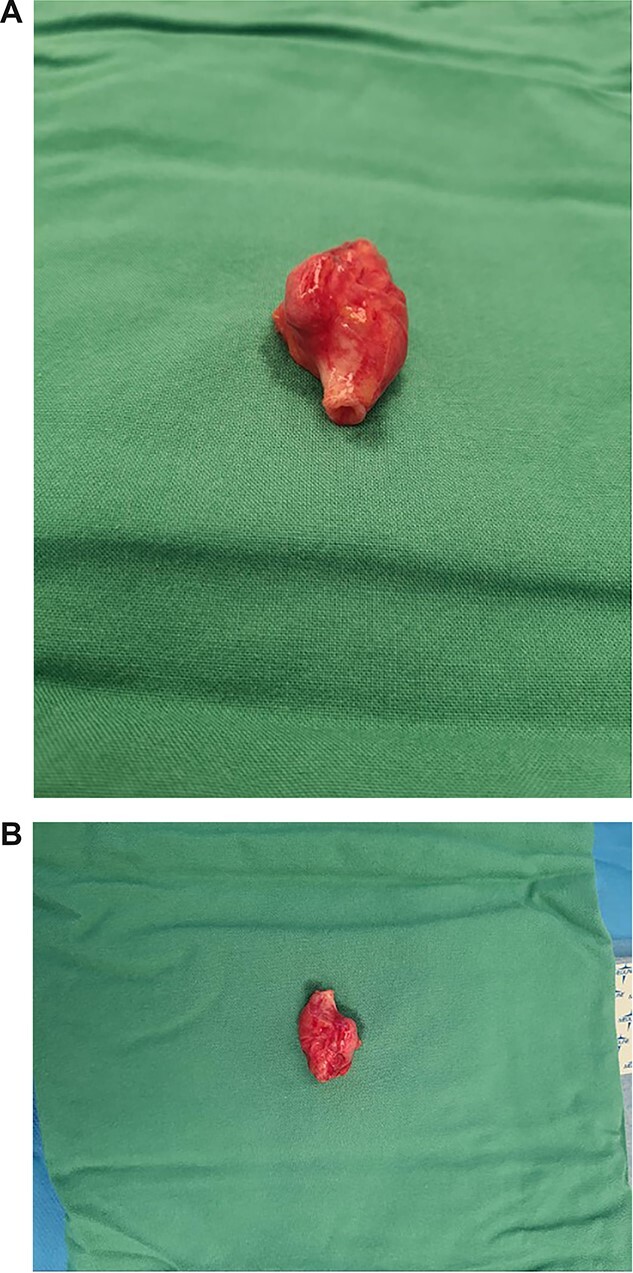
(**A**, **B**) Macroscopic views of resected ureteric leiomyosarcoma mass.

Case PD consented for a boari flap, psoas hitch, ureteric reimplant ± left nephroureterectomy. Following lithotomy and reflection of bowel, minimal mobilization was required to identify the ureteric mass located at the level of iliac bifurcation and appeared 2 cm in size. Good ureteric length was mobilized, and after excision of the mass, a tension free end-to-end anastomosis of ureter was performed without complication (Fig. 3).

**Figure 4:**
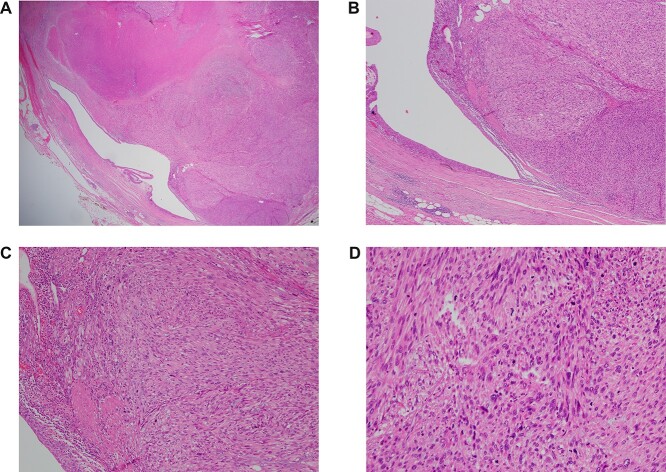
(**A**–**D**) H&E resection ×12.5, ×40, ×100 and ×200—ureteric lumen (left of picture) lined by stratified, transitional epithelium, with high grade leiomyosarcoma extending from the wall. Note the spindled appearance of the lesional cells, with marked nuclear atypia and mitotic activity. In the lowest magnification (×12.5), the area of necrosis is the ill-defined pink region in the top left hand corner.

Tumor histopathology confirmed a grade 3 leiomyosarcoma arising in the wall of the ureter and extending through the wall into the peri-ureteric connective tissue and to within 0.2 mm of the circumferential soft tissue margin. The ureteric margins were well clear (Fig. 4).

The patient recovered well post-operatively and was discharged home after removal of drain tubes and catheter. Repeat CT-IVP 5 months post-operation showed no evidence of tumor recurrence (Fig. 1B).

## CASE DISCUSSION

Around 20 reports of primary leiomyosarcoma of the ureter are in the literature, with most tumors arising from the ureter being transitional cell carcinomas [1, 2]. Smooth muscle tumors are more common in tumors of the bladder and kidney.

No clinical pattern has been associated with ureter leiomyosarcoma, however predominant presenting symptoms include flank or abdominal pain, urinary tract infection and palpable mass [3]. As with our case, diagnosis is based on the anatomapathological analysis with immunohistochemical markers, although hydroureteronephrosis on renal tract ultrasound and a soft tissue density mass on CT can be seen [1]. The mass may be visualized on ultrasound and has been described as cystic [2]. Ureter leiomyosarcoma lack a characteristic enhancement pattern on CT adequate to make a diagnosis [1]. Acute renal failure may occur secondary to ureteric obstruction [4].

Early literature reviews describe a short survival of reported cases; a 1988 review noted five patients died within 2 years of the disease out of seven existing cases with detailed follow-up following surgery [3].

Noting the aggressive nature of the condition, treatment of choice has been reported as total nephroureterectomy with en bloc resection of the tumor [3, 4]. Contrarily, some cases have been managed with tumor excision and ureteroureterostomy without signs of recurrence, similar to with our patient, though the case by Shastri recommended adjuvant radiotherapy post-operation, and in the case by Kolhartkar, the reason nephroureterectomy was not performed was due to the tumor arising from the patient’s only functioning kidney [4, 5].

No further cases of primary ureter leiomyosarcoma could be identified in the literature since the case reported by Shastri in 2016 to this date.

## CONCLUSION

It is important to report primary ureter leiomyosarcoma cases to assist further studies investigating the condition and the best approach to its treatment; in this case no malignant recurrence was observed 6 months following tumor resection and ureteroureterostomy.
